# Provider perspectives on the impact of COVID-19 on treatment of substance use and opioid use disorders among American Indian and Alaska Native adults

**DOI:** 10.3389/fpubh.2024.1356033

**Published:** 2024-06-05

**Authors:** Meenakshi Richardson, Katherine Hirchak, Kelsey Bajet, Mariah Brigman, Racquel Shaffer, Beverly Keyes, Karen Anderson Oliver, Frankie Kropp, Michael G. McDonell, Kamilla L. Venner, Aimee N. C. Campbell

**Affiliations:** ^1^Human Development, Washington State University Vancouver, Vancouver, WA, United States; ^2^Department of Community and Behavioral Health, Elson S. Floyd College of Medicine, Washington State University Spokane, Spokane, WA, United States; ^3^Promoting Research Initiatives in Substance Use and Mental Health (PRISM) Collaborative, Elson S. Floyd College of Medicine, Washington State University Spokane, Spokane, WA, United States; ^4^College of Education, Washington State University, Pullman, WA, United States; ^5^Independent Researcher, Tribal Lands, WA, United States; ^6^Independent Researcher, Seattle, WA, United States; ^7^Department of Psychiatry & Behavioral Neuroscience, College of Medicine, University of Cincinnati, Cincinnati, OH, United States; ^8^Department of Psychology and Center on Alcohol, Substance Use & Addiction, University of New Mexico, Albuquerque, NM, United States; ^9^Department of Psychiatry, Columbia University Irving Medical Center, Columbia University, New York, NY, United States

**Keywords:** American Indian and Alaska Native, COVID-19, medications for opioid use disorders, opioid use disorder, substance use disorders, harm reduction, access, health equity

## Abstract

**Introduction:**

American Indian/Alaska Native (AI/AN) communities are more likely to suffer negative consequences related to substance misuse. The COVID-19 pandemic exacerbated the opioid poisoning crisis, in combination with ongoing treatment barriers resulting from settler-colonialism, systemic oppression and racial discrimination. AI/AN adults are at greatest risk of COVID-19 related serious illness and death. In collaboration with an Indigenous community advisory board and Tribal leadership, this study explored AI/AN treatment provider perceptions of client-relatives’ (i.e., SUD treatment recipients) experiences during the pandemic from 2020 to 2022.

**Methods:**

Providers who underwent screening and were eligible to participate (*N* = 25) represented 6 programs and organizations serving rural and urban areas in Washington, Utah, and Minnesota. Participants engaged in audio-recorded 60–90 min semi-structured individual interviews conducted virtually via Zoom. The interview guide included 15 questions covering regulatory changes, guidance for telemedicine, policy and procedures, staff communication, and client-relatives’ reactions to implemented changes, service utilization, changes in treatment modality, and perceptions of impact on their roles and practice. Interview recordings were transcribed and de-identified. Members of the research team independently reviewed transcripts before reaching consensus. Coding was completed in Dedoose, followed by analyses informed by a qualitative descriptive approach.

**Results:**

Five main domains were identified related to client-relative experiences during the COVID-19 pandemic, as observed by providers: (1) accessibility, (2) co-occurring mental health, (3) social determinants of health, (4) substance use, coping, and harm reduction strategies, and (5) community strengths. Providers reported the distinctive experiences of AI/AN communities, highlighting the impact on client-relatives, who faced challenges such as reduced income, heightened grief and loss, and elevated rates of substance use and opioid-related poisonings. Community and culturally informed programming promoting resilience and healing are outlined.

**Conclusion:**

Findings underscore the impact on SUD among AI/AN communities during the COVID-19 pandemic. Identifying treatment barriers and mental health impacts on client-relatives during a global pandemic can inform ongoing and future culturally responsive SUD prevention and treatment strategies. Elevating collective voice to strengthen Indigenous informed systems of care to address the gap in culturally-and community-based services, can bolster holistic approaches and long-term service needs to promote SUD prevention efforts beyond emergency response efforts.

## Introduction

Harmful outcomes related to substance use disproportionally impact many American Indian/Alaska Native (AI/AN) communities compared to other racial and ethnic groups. This inequity exists even as there are higher rates of abstinence from certain substances (i.e., alcohol) among AI/AN adults compared with non-Hispanic Whites (1, 2). Higher rates of alcohol and drug use disorders are associated with historical trauma and political factors including colonization, forced removals, and efforts to eradicate culture and language, much of which impacts current social and economic challenges experienced by some Tribal communities (1, 3–10).

Structural factors contribute to elevated substance use risks which result in psychological distress and barriers to care that were further compounded during the COVID-19 pandemic (11–14). Among the general population in the US, the pandemic resulted in significant decreases in life expectancy, increased unemployment, economic instability, social isolation, depression, fear, and anxiety (15–18). From 2019 to 2020, rates of fatal drug poisonings among AI/AN adults surpassed other racial/ethnic groups, increasing 39% between 2019 to 2020 (19). These combined public health emergencies further strained AI/AN populations.

AI/AN communities led health care innovation during the pandemic, utilizing community driven strategies to attain high rates of COVID-19 vaccinations. They similarly innovated to continue to provide opioid treatment during the pandemic. To address the treatment needs of people experiencing substance use disorders (SUD), especially opioid use disorder (OUD), during the COVID-19 emergency, changes were made to meet service delivery demands (20). One study by Wendt et al. (21) assessing the impact of COVID-19 among Indigenous communities in the US and Canada, noted that the pandemic increased the flexibility in prescribing medications for OUD, dosing schedules, and access through the expansion of telemedicine. At the same time, providers also described reduced access to traditional Indigenous healing and medicine practices for addiction recovery, linking this to increases in substance use and mental health symptoms and decreases in overall well-being (21). Although regulatory flexibility was meant to promote greater availability and accessibility, little is known about the effectiveness of these changes among programs serving AI/AN communities.

To better understand the impact of these regulatory changes and the ways AI/AN communities navigated the service delivery landscape during the COVID-19 pandemic, we conducted semi-structured qualitative interviews with SUD treatment providers in AI/AN-serving programs. We assessed provider perspectives of the pandemic on the health, well-being, substance use and treatment access of client-relatives (throughout this paper we refer to service recipients as client-relatives). This term, ‘client relative’ was among the research team and chosen to highlight the relational and cultural connectedness rooted within kinship values. Hence, we uplift Indigenous worldviews to view clients as relatives. Our research question was: *From providers’ perspectives, what were the strengths and challenges client-relatives faced with respect to access and utilization of SUD treatment services, daily life and substance use during the first 2 years of the pandemic (2020–2022)?* Provider perspectives can help to inform future community-and culturally-specific public health responses and strategies across prevention, treatment, recovery, and harm reduction services.

## Materials and methods

### Community engagement and theoretical framework

Community Based Participatory Research (CBPR) strategies were employed throughout the study. CBPR is a bi-directional process that centers community knowledge and power-sharing between community and university partners (22). CBPR has led to improved health and social equity outcomes among Indigenous communities through the lens of Indigenous worldviews and systems of care (23–25). Interventions that seek to embody CBPR principles must also centralize cultural perspectives and community engagement (26). A national Collaborative Board (CB) was formed in 2019 as part of a parent project, consisting of more than 25 members with a range of backgrounds from across the country (27). To inform the present research, a sub-set of the CB provided consultation on the study design, research questions, and development of the interview guide. Further, we also connected with additional partners and developed relationships with Tribal leadership and behavioral health providers and practitioners at AI/AN-serving organizations and treatment centers across the Pacific Northwest.

### Positionality

Consistent with decolonizing methodologies and self-reflexive praxis, we acknowledge and highlight the identities and backgrounds of the researchers as they relate to the present study (28, 29). MR is a citizen of the Haliwa-Saponi Tribe and Asian Indian of Indo-Fijian descent, residing in the Pacific Northwest. She has walked alongside both rural and urban Indian communities for 8 years, providing health and human services, culturally informed programming, CBPR strategies, and Indigenous methodological approaches to prevention programming. KH is a descendant of the Eastern Shoshone Tribe/White, mixed European ancestry and has partnered with AI/AN communities in health justice research for more than 15 years. KB is a descendant of Filipino ancestry and immigrants, born in the Pacific Southwest. MB is a member of the Spokane Tribe of Indians. RS is a Cowlitz Tribal member descending from Chief Scenewa with mixed ancestry and has worked in the substance use disorder and medically assisted treatment fields for the last 5 years. KAO is White of mixed European ancestry and has partnered on research projects with Tribal communities and AI/AN researchers for 5 years. FK is White of mixed European ancestry and has collaborated on research projects with Tribal communities and AI/AN researchers for 15 years. MGM is Scotch-Irish and German American and has partnered with AI/AN communities on substance use treatment research and training for over 10 years. ANCC is White of mixed European ancestry and has partnered on research projects with Tribal communities and AI/AN researchers for 13 years.

### Participants

This study was approved by the Washington State University and Northwest Portland Area Indian Health Board Institutional Review Boards. Providers were recruited through advertisements (e.g., flyers) placed in partnering organizations, referral by partnering organizations or other agencies (e.g., social services, Tribal health, or others), word-of-mouth, and social media sites (e.g., Facebook). Six programs and organizations serving multiple Tribal and sovereign nations and urban areas in Washington, Utah, and Minnesota served as recruitment sites, a majority of which were from Washington state. Eligible participants were: (1) willing and able to provide informed consent (2) employed at an AI/AN-serving addiction program; (3) had direct client-relative contact (administrative or clinical); (4) aged 18 or older; and (5) fluent in English. Study data and individual participant profiles were collected and managed using REDCap (Research Electronic Data Capture) electronic data capture tools hosted at Washington State University (30, 31).

### Procedure

Interviews were conducted by the first and third author. Each participant completed one semi-structured interview. The interview guide included 15 base questions covering regulatory changes, guidance for telemedicine and HIPAA compliance, policy, procedures, staff communication, and perspectives on COVID-19 impact to their professional roles, as well as client-relatives’ reactions to changes implemented, service utilization, and changes in treatment modality. Examples include: “*What have you noticed about client-relative substance use since COVID-19 disruptions in March 2020* (e.g.*, increases, decreases in substance use and poisoning*)?” For a complete list of interview questions, see Supplementary Materials. The interviews ranged in duration from 60 to 90 minutes. Upon interview completion, participants were given a $100 gift card sent directly to their email. All interviews were audio recorded using secure Zoom video conferencing.

### Data analysis

The second and third authors independently coded 25% of the transcripts, applied thematic analysis to these data, and then came together to discuss preliminary codes and iteratively generate a codebook (32). Thus, the codebook was created per categorization and thematic analysis, from an in-depth review of the transcripts, and not *a priori* (33). The first and third author then coded all 25 transcripts. Thirty-six codes were finalized for review to address the current research question. The transcripts underwent an additional analysis informed by a qualitative descriptive approach (34–36). The coding procedure included four members of the research team. Two of which were the primary coders, a third for secondary review and the fourth for consensus. The inclusion of four coders ensured reliability, and the sample size, indicative of saturation (37, 38). Dedoose 9.0.85, a qualitative data analysis software, was used for all qualitative data management and analytic procedures.

## Results

### Overview

Thirty-six individuals were screened for participant eligibility. Of those screened, 8 were ineligible, with common reasons due to not being employed at an AI/AN-serving addiction program or not having direct client-relative contact; 3 did not respond to requests for scheduling; and 25 individuals consented to participate and completed interviews. Nineteen (76%) identified as female with ages between 30 and 60 years. Per recommendations of the CB, questions about race/ethnicity and other basic demographics were not asked due to privacy concerns. Participants held positions as clinical behavioral health practitioners, substance use disorder professionals (SUDPs), licensed mental health counselors (LMHCs), licensed clinical social workers (LICSWs), nurse practitioners (ARNPs), administrators, and other programmatic positions (Table 1).

**Table 1:** Brief overview of provider characteristics.

Provider characteristics		
Sample size	N = 25	
Age	M = 43.24	S = 8.12
Biological sex	Male 24%	Female 76%
Region type	Urban 84%	Rural 16%
Provider type	Clinical (e.g., ARNP, SUDP, LMHC) 64%

Five coded domains were identified from the data related to client-relative experiences during the COVID-19 pandemic, as observed by providers. These are detailed below and include: (1) accessibility, (2) co-occurring mental health, (3) social determinants of health, (4) substance use, coping, and harm reduction strategies, and (5) community strengths.

### Accessibility

Providers highlighted several challenges related to client-relatives access to SUD treatment services during the pandemic, including general lack of access to care due to program closures, navigating changes in service flow leading to limited availability and capacity of providers to meet growing need for treatment services. In addition, providers described that the limited number of pharmacies available to client-relatives were inundated during the pandemic and accessing prescription medications was severely delayed. Providers shared client-relatives’ collective expressions of stress and frustration due to these regulatory challenges impacting their capacity to provide services, but flexible in response to the public health emergency. Despite understanding the need for changes to protect public health, providers described limitations on continuity of care which carried well into the pandemic. A provider expanded by stating:

…some people were very receptive [to the changes in care, while from] some people there was a lot of pushback and completely disengaged. I think that was a challenge [and] for some people that change in the way we delivered services, and just trying to get it all together was really disruptive in their recovery. There were people that had been maintaining abstinence that relapsed and may have not engaged in services the same way…some people really liked doing telemedicine… I can’t say it’s one way or the other. I mean, it was a disruption the way that it was for everybody.

Moreover, providers overwhelmingly stated how the pandemic placed an additional burden on top of an already under-resourced system which further strained SUD service delivery. Providers described that integrated group sessions were a large component of treatment services and in some cases an accompaniment to individualized therapy sessions or talk-therapy. Although due to the pandemic, the transition to alternative treatment delivery formats (e.g., telemedicine) was generally experienced as positive, there were difficulties in facilitating group sessions and engaging client-relatives, especially when a client-relative was also experiencing complex traumatic issues. The following quote described challenges to virtual group work, including mutual support groups:

…groups became very restricted and the services were obviously crippled as a result …. Things were shut down [and] it was quite a while before … community support groups like [Alcoholics Anonymous] and [Narcotics Anonymous] and [Cocaine Anonymous] opened back up. They did move to online, but I don’t know that that carries the same … [the] connectedness that going to an in-person 12-step meeting has versus via Zoom. I think that’s really impersonable, and if you don’t know anybody, if you’re just now trying to enter into recovery and stop using, walking through the door is already tough but not having that human connection, I think it makes it extremely tough … to wanna stick around.

### Co-occurring mental health

A majority of the sample reported an exacerbation of co-occurring mental health concerns among client-relatives. Mental health challenges arose due to increased stress related to school closures and parenting with limited resources. Reductions in social and structural supports (e.g., school) and subsequent impact on mental health left some providers feeling ill-equipped to support client-relatives. Providers described the exacerbation of trauma-related mental health symptoms among client-relatives and the overall impact of the pandemic on Indigenous communities, such as the compounding nature of post-traumatic stress disorder (PTSD), increased anxiousness and intergenerational trauma colliding with service closures due to the COVID-19 pandemic. Providers addressed these needs and how they attempted to keep up with and in some cases expand care, while also navigating the broader healthcare system:

We've seen a big uprise in the need for… mental health services, and so our department just recently started a co-occurring program where now we can ... start assessing for ... mental health. Prior to that, we were having just to refer out to our mental health department here at the Tribe. So there's definitely been a higher need, for sure ... and that’s something ... that our mental health department has grown, and our co-occurring services we're starting to do are definitely taking off … [as the] need has grown….

Delivering services during the pandemic were also considered within the context of historical and intergenerational trauma. Providers specifically addressed this and the way they navigated client-relative care:

I think that … working with Native communities … there is inter-intergenerational trauma that is always in the room and not often recognized by the client. It's like… maybe at first you recognize that it's something outside of you creating [hardships], and then eventually you think it's just something wrong with you. … And folks don't understand that … they're carrying this mass trauma, and that that is actually what has made their lives difficult.

Providers reported the cultural impact of grief and loss for many Indigenous communities throughout the pandemic as significant, making direct connections to historical loss and trauma perpetuated into the present day and heightened further during the pandemic. It was shared that many client-relatives lost loved ones during this time and the AI/AN population at large are experiencing collective trauma as a result of the COVID-19 pandemic, amplified by both historical and contemporary traumas. The inability to conduct ceremonies and engage in cultural, spiritual, and cleansing practices affected the ways in which client-relatives were able to regulate emotions and engage in their own treatments:

… [there is] disconnect [ion] … a lot of my elder clients, they were very safe and stayed home. But you could tell it was takin' a toll on their mental health as far as not being able to participate. And especially with… the culture and [individuals] not being able to participate in community dinners for, [and] I'm speaking for several Tribes… not being able to, powwows and, smokehouse… canoe journey or, blessing of the fleet, or clam bakes. Like, these are events that, clients look forward to. And not only clients but, you know [also us as providers] … it's a privilege. I've worked for the Tribe for seven years and I've been honored to be able to participate in these events… that's where I meet a lotta family and … they struggled with not having that spiritual [connection] ….

### Social determinants of health

Providers spoke about the impacts of COVID-19 on the community through the lens of social structural drivers of health. Many noted that the negative impacts were detrimental to their client-relatives’ recovery. Specifically, providers discussed barriers related to employment, housing, and transportation. Providers addressed these issues with client-relatives concurrently with navigating the pandemic and their recovery. Providers also spoke to the prolonged stress linked to the pandemic, including long-term stressors associated with transportation:

So, transportation was such a big mess [throughout] COVID [and] continues to be a mess. I hope that that was really highlighted for people, and maybe, could be a focus for Tribal government in terms of funding moving forward ‘cause we can anticipate that there will be another epidemic or crisis where transportation’s gonna be crucial.

One provider noted that their organization provided transportation, although they were unable to bill for this service outside of medical appointments or to pick up prescription medications. Client-relative challenges regarding transportation included lack of personal vehicles, limited public transportation, distance, and financial constraints in purchasing gas or public transportation passes. Some client-relatives noted that because they did not have a personal vehicle, they became reliant on a family member or friend for transportation, which caused some difficulty in coordinating scheduling.

Providers spoke to the housing crisis and the inequitable access to stable housing for client-relatives, noting that experiences of houselesness were also exacerbated due to the pandemic. Providers described the necessity of stable housing for treatment effectiveness and overall health. The following quote describes the relationship between housing instability and limited availability of higher levels of care for substance use:

… just that incongruence within themselves where they want help, but then their addiction is very strong in them… [causing] difficulty in getting into inpatient programs, which seems like it was worse because there was more people … going back to their using and then wanting help and not being able to get in… we’ve had a client here, sleeping in their car on our parking lot for months… I think it took two and a half months to get him [housing and other support services] … and that’s just one in particular that I can think of, long waits to get in, exceptionally long.

Additionally, providers address the interwoven nature of in-person cultural activities integrated into SUD services such as drumming circles, traditional medicine services, beading and art groups, sweat lodges, and other healing ceremonies, which are imperative to client-relatives’ well-being and treatment progress. These services, either could not be offered or were limited in offerings during the pandemic. Providers expressed that the lack of cultural connection and integrative components of traditional health severely impacted continuity of care.

### Substance use, coping, and harm reduction strategies

Most providers reported seeing an increase in the use of fentanyl, methamphetamine, cannabis, and alcohol directly related to the pandemic. One participant provided the following statement in response to increases in substance use:

I think that [substance use has] increased. I think that people that were stable, that when the groups [and] society shut down … there was really nothin’ to do, nowhere to go for people and I think that boredom set in, and stress set in, and fear set in, and I think that a lotta people relapsed as a result. I think that people that were coming through the doors trying to enter into recovery and had virtually no services available for them, [they] struggled. I really think [the pandemic] set our field back quite a bit as a result.

In addition, there were individuals that shifted the type of substances used but providers did not necessarily identify that shift as an increase:

…within the last year, we’ve seen the drug of choice change a lot. Like right now, we’re experiencing a lot more fentanyl use … [among] youth and adults, especially … Alcohol as well as well, where prior to COVID … it seemed to be more marijuana [and] DUIs. That’s something, we can track, too, where it seems like alcohol definitely was on the uprise, and then right now, like within the last six months, at least in our area, we’ve seen fentanyl use just spike where [it] was not a thing pre-COVID, at least not to where we were seeing it on a consistent basis.

The changes in services due to the pandemic was a concern for providers and seen as impacting how client-relatives might utilize less healthy ways to cope to manage emotion dysregulation and limited social connection. A provider outlined this association between service availability and choice of coping strategies:

[Some clients] have this mentality of, like, I, [as the provider] can fix all your problems for you. Like, that’s not real, right? People know how to regulate their own bodies. People know how to self-medicate, and that’s a coping skill, whether people wanna admit it or not. Using drugs is a coping skill. My goal in my career is just to give you something that maybe isn’t gonna poison you, just as an option, and you can choose it or not …. But, you know, you are doing a coping skill. You do know how to regulate your body. You are smart, and you’re resourceful and resilient …so we need fully integrated treatment, that is so essential and vital because—it’s about showing somebody that there is a different way, and they can choose something different. And, at the end of the day, they gotta make that choice.

Harm reduction strategies were seen as a way to mitigate risks associated with increased substance use. Although Naloxone training and education was provided for healthcare providers and peer support workers at their respective organization, the impact of COVID-19 on policies, procedures, and social distancing, as well as staff fidelity to uphold such processes, influenced the successful implementation of these strategies for client-relatives. Several providers alluded to the availability of Naloxone as beneficial and in alignment with their support of harm reduction strategies, but with noted challenges. One participant noted that: “There’s not very much access to Naloxone training, unfortunately. So I try to educate people, as they come in … lots of fentanyl.” Providers specifically discussed the inconsistency in implementing Naloxone distribution protocols:

I know that the procedure is the person comes to the back door, rings the bell, asks for one, and, you know, a nurse or whoever’s on will give them one. And, I’m just like, “Is anyone havin’ a conversation with these people, or are they just handin’ the bags out and-and shuttin’ the door?” … there needs to be a conversation. I know that [with] the needle exchange programs, it’s not like I’m [just] going around [handing them out]. They [are available] in a specific location, [and we provide outreach] you know, interact with people and get to know them and counsel them a little bit to try and initiate some kind of change.

Identifying access and use of Naloxone is just one harm reduction strategy. Providers emphasized that strategies ultimately need to be client-relative centered, coordinated, and holistic to ensure that communities are as safe and healthy as possible.

### Community strengths

It was vital to providers to share community strengths and the specific ways in which their respective organizations came together with the goal of relationality and healing while navigating the challenges of the pandemic. Providers detailed that culturally specific programming (e.g., drumming, beading, feasts) that had previously been integrated into treatment services were halted or transitioned to an online format. Overall, providers spoke to the continuation of virtual cultural services as important to social connection. Through the grief in loss, substance use, and impact on communities, the shared focus on community and cultural connectedness was described as pivotal in promoting resilience, recovery, and prevention. Providers described how their organizations and communities were able to access hope and lean on cultural beliefs, values, and practices. A provider summarized this sentiment in the following way:

What I love ... about the clinic is that, even though we were all stuck in this, like, fear of COVID and not knowing and a medical emergency pandemic, we were still reminding the community, let’s go back to our basics. Let’s go back to what we know and what comforts us. So it was really awesome to be part of that, to see it. So and even now, we’re still offering our traditional medicine, so I really just wanted to also mention that too cause that’s something I also saw outta the pandemic that was just really inspirational, and I feel like it really lifted the spirits of the community here, since, you know, we’re such a small little area … it’s just like the one lifting of one person’s heart and it spreads around, you know. So it was really cool …. I’m just really happy to be still here, still part of it, and working through it. So hopefully things will get better. I know that they probably could never go back to the way they were, but, I think that it’s gonna make us all into more resilient people, I hope.

## Discussion

In this qualitative community-based participatory research study, we examined SUD providers’ perspectives on the challenges experienced by client-relatives receiving substance use treatment services for opioid use disorder during the COVID-19 public health emergency. Overall, providers observed exacerbation of mental health symptoms and other comorbidities among client-relatives due to social structural challenges and reduced access to both AI/AN traditional healing practices and substance use services. Findings add to the little representative research on AI/AN serving organizations’ response to, and perceptions of, client-relative experiences and needs for SUD treatment services during a national public health emergency. Findings help inform preparation and planning for the advancement of public health strategies and service delivery.

Accessibility of services for client-relatives was a huge concern for providers. Although providers noted the benefit of the programmatic and regulatory changes in SUD services related to telemedicine for client-relatives, barriers remained. Similarly, Nesoff et al. (39) conducted a cross-sectional analysis among non-AI/AN adults accessing or in need of treatment, to quantify the extent of policy uptake and the number of people with SUD impacted by policy changes at the state level across the U.S. Nearly half a million people who were accessing treatment before the pandemic had limited or no access to treatment at the onset of the pandemic. Providers in our study noted that there were minimal services prior to the pandemic, and that this limited access was intensified during the pandemic and led to increases in substance use and poorer mental health. A combination of strategies to increase availability, access, and engagement into care are necessary. In May of 2023, the Drug Enforcement Administration (DEA) announced an extension to the flexibilities adopted during the COVID-19 public health emergency including those related to telemedicine, protocols for controlled prescription medications (e.g., length of prescriptions), consultation and examinations (40) which is promising news for AI/AN communities who continue to manage high rates of poisonings and social structural barriers (e.g., transportation). Although regulatory flexibility could not alleviate all challenges, it was seen as generally positive, with hopes of seeing such flexibility as common practice, rather than a temporary emergency response effort.

There was also consensus among participants in our study that, among many AI/AN communities, health inequities were directly tied to historical, intergenerational, and ongoing traumas. Ramos et al. (41) address this link among AI/AN populations in California, investigating experiences of substance use, homelessness and treatment seeking. They found that services needed to be culturally grounded and responsive to the needs of AI/AN people experiencing homelessness to reduce substance use. The COVID-19 pandemic brought about collective trauma among AI/AN communities, resulting in an amplification of health and social disparities. Moreover, SUD services should better integrate the role of historical and social contexts to understand and serve AI/AN communities more effectively. Intervention strategies that centralize the integration of traditional approaches and cultural activities that consider both the historical and contemporary traumas and challenges that AI/AN communities’ face with respect to SDOH, behavioral health, and SUD outcomes are therefore needed (42). This is especially relevant in the context of national and global public health crises like drug poisonings and COVID-19. Providers emphasized the integral component of cultural services in treatment and community connectedness that are unique to the AI/AN population. The inclusion of traditional medicine, songs, drumming, language teachings, beading, and arts are imperative to the recovery and healing of client-relatives and support health relationships. Moreover, the minimal resources and funding allocated for traditional health and cultural healing practices as part of SUD services with regards to Medicaid, Medicare, and private insurance reimbursement impact the extent to which culturally relevant and traditional care can be provided. There is a need for culturally responsive providers, and building capacity for service providers who identify as AI/AN and those with lived experience (43, 44).

Some common limitations should be considered within the current work. Although the sample was not restricted to a specific geographic area of the U.S., most providers were employed by, or served, client-relatives from Tribal communities in the Pacific Northwest. While the challenges reported regarding accessibility and substance use are represented in multiple populations, findings are not necessarily generalizable to all Tribal communities. Further, these challenges are from the perspective of providers and not client-relatives themselves. In addition, although appropriate due to the safety protocol and regulations throughout the COVID-19 pandemic, conducting interviews virtually rather than in-person may have altered interpersonal and social dynamics impacting engagement and responses. The inclusion of providers was dependent upon the availability of providers and respective approvals in participation per respective treatment services or agencies, and participant inclusion was not specific to provider type (e.g., ARNP, LMHC). Per the guidance of the CB, community voice was prioritized in the decision to not collect racial, ethnic, or biological sex demographics of participants. Strengths of the research include the comparatively large sample size and interview content domains that considered treatment providers’ efforts to deliver services that centered history, culture, healing, resilience, and social connection for client-relatives during the pandemic.

The implications of these findings suggest expanding capacity and workforce initiatives within AI/AN populations to equip AI/AN community health workers and mental health practitioners to provide substance use treatment and recovery services that are grounded in local cultural and traditional practices and that can support social drivers of recovery success such as connectedness, economic instability, houselessness, transportation, and integrated behavioral health services (45). Investigating and expanding culturally informed harm reduction strategies inclusive of Naloxone and Narcan through AI/AN community-led programming and Indigenous research methodologies that center stories and cultural strengths is important (46). Such efforts should not just be limited to the pandemic period but used to build the capacity of AI/AN-serving treatment and prevention programs.

## Conclusion

Findings highlight SUD treatment provider perspectives on the impact of the COVID-19 pandemic on client-relatives’ experiences in accessing treatment, managing stressors, and fluctuations in substance use and mental health. Findings also illuminate experiences of resiliency and hope and the ways in which culturally specific community and public health related response strategies, preventative approaches, and long-term service needs can be leveraged to promote well-being among AI/AN adults experiencing SUD or who are in recovery. These strategies may include Tribal best practices, expanding harm reduction strategies, building community and systems capacity, and providing adequate funding directly to AI/AN-led and serving programming addressing SUD.

## Data availability statement

The datasets presented in this article are not readily available because data sharing agreements restrict data access to only authorized researchers. Requests to access the datasets should be directed to meena.richardson@wsu.edu.

## Ethics statement

The studies involving humans were approved by the Washington State University (#18604) and Northwest Portland Area Indian Health Board Institutional Review Boards (#1743728-3). Tribal Council approvals and/or data sharing agreements were received from several Tribal Nations, however we do not disclose the names of the sovereign nations due to established data sharing agreements. The views expressed in this paper do not necessarily reflect that of NIDA CTN and partnering communities. The studies were conducted in accordance with the local legislation and institutional requirements. The participants provided their written informed consent to participate in this study.

## Author contributions

MR: Conceptualization, Data curation, Formal analysis, Methodology, Software, Validation, Writing – original draft, Writing – review & editing. KH: Conceptualization, Data curation, Formal analysis, Funding acquisition, Investigation, Methodology, Project administration, Resources, Software, Supervision, Validation, Writing – original draft, Writing – review & editing. KB: Data curation, Formal analysis, Methodology, Software, Writing – review & editing. MB: Data curation, Methodology, Writing – review & editing. RS: Conceptualization, Writing – review & editing. BK: Conceptualization, Writing – review & editing. KO: Conceptualization, Writing – review & editing. FK: Conceptualization, Writing – original draft, Writing – review & editing. MM: Conceptualization, Writing – review & editing. KV: Conceptualization, Data curation, Funding acquisition, Investigation, Methodology, Writing – review & editing. AC: Conceptualization, Data curation, Funding acquisition, Investigation, Methodology, Writing – review & editing.
