# Supplementary material for: Provider perspectives on the impact of COVID-19 on treatment of substance use and opioid use disorders among American Indian and Alaska Native adults
Source: Front Public Health. 2024 Jun 5;12:1356033. doi: 10.3389/fpubh.2024.1356033 (PMC11186410; doi:10.3389/fpubh.2024.1356033)
Supplement: Supplementary file 1 [file Data_Sheet_1.docx]

**Guiding Questions for Providers**

Policy and Regulatory Changes

1. “How were regulatory changes related to providing addiction services (e.g., guidelines for telemedicine, dosing, HIPAA compliance) communicated to you and your program?” *To interviewer: Share screen via Zoom and show the policy table in appendix to the individual or read list of changes if interviewing over the phone*.
2. “Describe the policy changes, if any, that were adopted by your program (e.g., temporary, emergency revision to the delivery of medications, telemedicine, etc.)?”
3. “Can you describe how your program was funded during this time (e.g., CARES Act, IHS, SAMHSA COVID-19 funding)?”
4. “Were there challenges related to funding or reimbursement?”
5. “Who does your program look to for strategies related to addressing the impact of COVID-19 on service delivery? (e.g., Tribal government, Tribal programs, IHS, SAMHSA, state, federal, etc.)”
6. “Please describe all the changes made to program policy as a result of COVID-19?”

*If not addressed in their response follow-up questions would include:*

“How was the need for PPE addressed in your organization? Were these made available to staff/consumers?”

“Did your program offer alternative modes for services (e.g., telehealth), screen consumers for COVID-19, impacts on supply chain of medications)?”

“Availability to provide ‘mobile treatment’ services (e.g., mobile treatment going to peoples’ homes, delivering medications, transportation to group)?”

“Availability of telehealth devices (e.g., webcams, stable internet connection)?”

“Availability of space for social distancing at your organization?”

“Vaccines/vaccine hesitancy”

1. “How have your services changed because of COVID? (e.g., individual and groups, shorter vs longer, more or less clinical time, etc.)?”

Adoption or Discontinuation of Policy/Regulatory Changes

1. “How did your program go about getting regulatory and policies up and running? What did the process look like? What were the challenges related to implementation?”
2. “What would need to change for your program to continue offering services in this new format (e.g., telemedicine visits, relaxed medication dosing protocols) beyond the current COVID-19 public health crisis?”
3. “What has been the staff acceptability of the policy and regulatory changes? Which changes is your program likely to keep? What have you liked, what do you hope to continue to implement?”

Impacts of COVID-19 on Consumers and Providers

Provider Perspectives on Patients:

1. “How did staff perceive consumer responses and reactions to regulatory changes?”
2. “What consumer experiences or characteristics have you observed/heard about that may have contributed to changes in how you or your agency engage the consumer in services and how they utilize treatment (e.g., consumer transportation issues)?”
3. “What have you noticed about client substance use since COVID-19 disruptions in March, 2020? (e.g., increases, decreases in substance use and overdose?)”
4. “What have you noticed about client mental health and mood symptoms?”

Provider’s Own Experience:

1. “What kinds of challenges have you experienced personally due COVID-19? (e.g., stress, distress, depression, anxiety, or insomnia)? If there have been challenges, how have these challenges impacted your ability to provide services?”
